# Salivary Cytokines in patients with Head and Neck Cancer (HNC) treated with Radiotherapy

**DOI:** 10.4317/jced.56318

**Published:** 2019-11-01

**Authors:** Sara Principe, Valentina Dikova, José Bagán

**Affiliations:** 1 PhD Candidate, Faculty of Medicine and Dentistry, University of Valencia, Fundación Investigación Hospital General Universitari (FiHgU) Valencia, Spain; 2 Service of Stomatology and Maxillofacial Surgery, Hospital General Universitari de Valencia, Faculty of Medicine and Dentistry, University of Valencia, Spain

## Abstract

**Background:**

Head and Neck cancer (HNC) is the sixth most common cancer in the world. The 5-year survival rate depends on early diagnosis and appropriate therapy. Typically, late diagnosis requires not only surgical intervention, but also radiotherapy treatment combined or not with chemotherapy. Ionizing radiation is known to increase the expression of a number of cytokines involved in inflammation, wound healing and toxicity areas. Salivary cytokines have promising features to be used as biomarkers for disease screening and outcome prediction in this malignancy. The aim of this article is to analyze the role of salivary inflammatory response elements in HNC patients treated with radiotherapy, their modulation and association with the treatment outcome.

**Material and Methods:**

A bibliographical search was performed on Pubmed, Cochrane and Embase using the following keywords: cytokines, saliva, head and neck cancer, radiotherapy. The cut-off point for the research were scientific papers published over the last 10 years. After a two-step selection process, 15 articles were identified and subjected to review.

**Results:**

Radiotherapy treatment was shown to influence a number of salivary cytokines. A trend towards a growth of IL-1β, IL-6, IL-8, MCP-1, and TNF-α levels was observed and it correlated with the irradiation dose.

**Conclusions:**

The analysis of salivary cytokines could be a useful biomarker for predicting radiotherapy outcome in HNC. However, large-scale investigations are required to validate the use of these cytokines in predicting and diagnosing HNC, as well as evaluating the response to the treatment.

** Key words:**Cytokines, saliva, head and neck cancer, radiotherapy.

## Introduction

Head and Neck cancer (HNC) is a complex and heterogeneous malignancy, encompassing a variety of tumors that originate in the hypopharynx, oropharynx, nasopharynx, larynx, lips, or oral cavity. The disease group is associated with a distinct epidemiology, etiology, and the required therapies are also different. Worldwide, it represents the sixth most common neoplasia and accounts for 6% of all cases, being responsible for approximately 1-2% of tumor deaths (1). Despite all the diagnostic and therapeutic advances, the 5-year survival rate of patients with HNC remains relatively unfavorable, around 50%.

Alcohol and tobacco use are among the most common risk factors for HNC (2), although high-risk human papillomavirus (HPVs) infection, especially type 16, has been implicated in the pathogenesis of HNC arising from the oropharynx (3). Treatment approaches can include surgery, radiotherapy (RT), chemotherapy (CT), targeted therapy or a combination of them. The selection of unique or combined modality treatments is based on considerations such as disease control probability, anticipated functional outcomes, tumor resection and the patient’s general condition (4). Survival and cure benefit from early diagnosis and appropriate therapy. However, late diagnosis usually requires surgical intervention and chemo-radiotherapy treatment.

Ionizing radiation is known to increase the expression of a number of cytokines involved in inflammation, wound healing and toxicity (5). Cytokines are intercellular signaling proteins that play a role in regulating growth, cellular proliferation, angiogenesis, and tissue repair. They also have a function in immune responses to infection, injury and inflammation (6). Cytokine levels are generally kept within a specified range and time; if not properly maintained, they can lead to induction of tissue damage. Cytokines have also been investigated in saliva as potential protein biomarkers of HNC (7)(5). In particular, various inflammatory and angiogenic cytokines have been proven to be increased in the saliva of tumor patients (8).

The most frequently analyzed salivary proteins are EGF, interleukin 6 and 8, and to a lesser extent other NFκB-derived members such as TNFα or interleukin 1β, interleukin 4 and 10, and VEGF (9). However, several oral and systemic conditions, including periodontal disease, Sjögren syndrome, and rheumatoid arthritis can also give rise to increased levels of inflammatory proteins in saliva (10). Evaluation of tumor and normal tissue in irradiated areas using biopsies or invasive techniques for correlative assays may be limited with respect to healing during and after therapy. Sampling of saliva is feasible and could provide a non-invasive method to prove the local effects of ionizing radiation on tumor and normal tissue. Salivary cytokines have promising features to be used as biomarkers for disease screening and outcome prediction in this malignancy.

The majority of saliva studies have focused on relative levels of cytokines in saliva between HNC patients and healthy individuals (7). However, changes in saliva from pre- to post-treatment have not been extensively explored due to the destruction of salivary glands and subsequent xerostomia with conventional radiation. This impasse changed with the emergence of salivary gland-sparing radiation that allows saliva recovery (11).

As the use of gland-sparing, intensity-modulated radiation therapy (IMRT) increases, Russo *et al.* in 2016 (8) investigated a panel of cytokines in whole saliva from HNC patients, pre- and post-treatment. Even though most studies mention elevated concentrations of candidate tumor markers in saliva, this is not true for all of them, as reductions in peptide expression have also been reported (9). Dealing with this, the majority of investigations have targeted EGF. This growth factor not only positively influences cell proliferation, but its levels also positively correlate with the invasive behavior of oral cancer cells. Indirectly, the decrease in EGF during and after radiotherapy of head and neck cancer patients could be the cause for impaired wound healing in the oral cavity and adjacent tissues, due to the fact that the salivary glands are often located within the beam projection (9).

Screening salivary cytokine levels could provide a useful approach to the identification of biomarkers for predicting radiotherapy outcomes. When applied to a high-risk population such as HNC survivors, a saliva-based test utilizing a panel of biomarkers for HNC could provide an accurate, non-invasive and relatively inexpensive monitoring method (7).

The aim of this article is to make an overview of salivary cytokines modulation and their association with radiotherapy outcomes in HNC patients.

## Material and Methods

A literature review was performed on scientific articles on HNC, salivary cytokines and radiotherapy treatment. The research included studies published on Pubmed during the past 10 years. This was then supplemented by a new search using the same key words in the following database: Cochrane and Embase.

The inclusion criteria for selecting articles were as follows:

- studies that focused on biomarkers for HNC diagnosis and/or treatment outcomes related with radiation or chemoradiation therapy.

- studies that included patients with HNC and OSCC (Oral Squamous cell carcinoma).

- studies that used saliva for the analysis of these biomarkers.

Studies that used different biological media such as blood, serum or body fluids instead of saliva as potential media diagnostics and/or to monitor HNC patients, and also articles that reported associations between saliva and cancer in experimental studies (in vitro or in vivo animal studies) were excluded.

The references cited in the selected papers were also checked for any incremental references that may have inadvertently been omitted during the electronic database searches.

The study selection was accomplished in two phases. During the first phase, a selection of the articles that appeared to meet the inclusion criteria based on their titles and abstracts was performed. In the second phase, full articles were evaluated and the final selection of 15 papers was based on the full text of the publication.

For all the included articles, the following information was recorded: year of publication, author(s), country, sample size, patient age, study methods, list of cytokines analyzed, and main conclusions.

## Results

The studies were conducted in six different countries: mostly in the United States, and the rest in Brazil, Canada, Croatia, Iran and Italy. The salivary cytokines were assessed by different methods, the most common being the enzyme-linked immunosorbent assay (ELISA), followed by the multiplexed immunobead-based assay technology. An overview of the summarized data regarding the selected articles is provided in  Table 1.

**Table 1:**
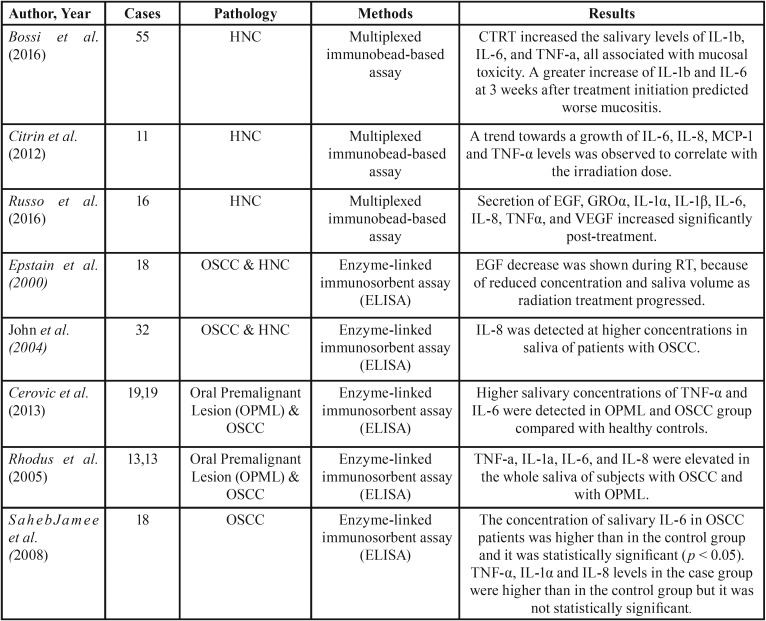
Summary of descriptive characteristics of included studies.

Bossi *et al.* in 2016 (12) followed the concentration, the modulation and the association of salivary cytokines with oral mucositis severity in HNC patients subjected to chemoradiation therapy (CTRT). The study was a prospective observational study and included 55 individuals diagnosed with locally advanced HNC. The levels of 13 cytokines were quantified before and after treatment. Overall increase was observed while the therapy was ongoing, especially in patients with severe mucositis. CTRT seemed to cause a significant growth of Interleukin 1β and 6 (IL-1β, IL-6) and tumor necrosis factor-α (TNF-α), all positively associated with the severity of mucosal toxicity.

Citrin *et al.* in 2012 (5), in a pilot study, explored the feasibility of using an ophthalmic sponge (Merocel) to sample saliva and to evaluate cytokine concentration in HNC patients undergoing CTRT. The results support the hypothesis that cytokine levels increase during the course of radiotherapy. Besides, their amounts vary depending on the proximity to the tumor irradiation area. Salivary levels of IL-4, IL-6, IL-8, EGF, MCP-1, TNF-α, and VEGF were found to be augmented in high dose radiation region when compared to the low dose one. A trend towards a growth of IL-6, IL-8, MCP-1, and TNF-α levels was observed to correlate with the irradiation dose.

Russo *et al.* in 2016 (8) showed a post-treatment increase in multiple cytokines in stimulated whole saliva from patients with HNC. Eight cytokines — TNFα, EGF, GROα, IL-1α, IL-1β, IL-6, IL-8, and VEGF — were analyzed and quantified concurrently in a multiplex assay. GROα, IL-1α, IL-1β, Il-6, IL-8, TNFα, and VEGF increased pre- to post-treatment.

Epstain *et al.* (13) showed that saliva volume and total EGF output decreased significantly in the first weeks of RT treatment and remained reduced throughout the therapy. In a previous study by the same author, with 18 head and neck carcinoma patients, EGF in saliva was also shown to decrease during RT due to reduced concentration and reduced saliva volume as radiation treatment progressed (14).

Previous studies have also demonstrated that some pro-inflammatory cytokines such as TNF-α , IL-1α, IL-6, IL-8, granulocyte-macrophage colony-stimulating factor receptor (GM-CSF) and VEGF can be found in significantly elevated levels in the local environment of OSCC (15).

In 2015 Guerra *et al.* (16) in their systematic review and meta-analysis evaluated the diagnostic value of salivary biological markers in the diagnosis of head and neck carcinoma. IL-8 was the most commonly assessed biomarker, analyzed in five studies. Among these studies, St. John *et al.* (17) obtained the best diagnostic capability results. This study reported that IL-8 was detected at higher concentrations in the saliva of patients with HNC (*P* < 0.01), and the findings suggest that IL-8 may hold promise as a biomarker for oral cancer.

The study performed by Cerovic *et al.* in 2013 (18) showed that TNF-α and IL-6 cytokines were detected in the whole saliva of 57 patients who were examined between 2008 and 2010 at the Department of Oral Medicine and Department of Oral and Maxillofacial Surgery of the Medical Faculty, University of Rijeka, Croatia. They were divided into three groups: 19 patients with oral premalignant lesions (OPML), 19 with OSCC and 19 healthy control volunteers. Results proved the patients with OPML and OSCC to have higher salivary cytokine concentrations compared with healthy controls.

Rhodus *et al.* in 2005 (15) reported increased concentrations of salivary IL-6 and TNF-α in oral cancer patients and in patients with oral precancerous lesions, while St John *et al.* (17) found no significant differences in salivary IL-6 in oral cancer patients compared to healthy controls.

The pathological activities of TNF-α have attracted much attention. For instance, although TNF-α causes necrosis in some types of tumors, it promotes growth of other types of tumor cells. High levels of TNF-α correlate with increased risk of mortality. These results indicate that increased salivary concentrations of TNF-α and IL-6 reflect local production of these cytokines in cancer tissue (18).

In 2008, SahebJamee *et al.* (19) confirmed that the concentration of salivary IL-6 in oral squamous cell carcinoma patients was higher than in the control group and it was statistically significant (*p* < 0.05). The concentration of salivary TNF-α, interleukin 1α and 8 in the disease group was higher than in the control group but it was not statistically significant (*p* > 0.05). These results show that more studies are needed to accept the utility of these cytokines in the prediction or diagnosis of oral squamous cell carcinoma and the evaluation of treatment.

Finally, Sahibzada *et al.* in 2017 (20) in their review also suggested that there is an elevation of IL-6, IL-8, IL-1 and TNF-α in OSCC and other oral neoplastic lesions because of their characteristic features, such as pro-angiogenesis and pro-inflammation effect which, in turn, has a diagnostic value.

## Discussion

The result of this literature review indicates that radiation or chemoradiation therapy can influence a number of salivary cytokines and, consequently, the analysis of salivary cytokines could be a useful biomarker for predicting radiotherapy outcome in HNC. Several studies reported increases in salivary IL-8, TNF-α, IL-1, IL-6 in HNC compared to controls but did not quantify changes post-treatment. A surprising finding observed by Russo *et al.* (8) was the post-treatment increase in cytokines. The study quantified cytokines pre- and post-treatment. Secretion of IL-1α, IL-1β, IL-6, IL-8, TNFα, and VEGF increased significantly post-treatment but EGF showed no changes. This may be related to the response to treatment.

Another factor to be considered is the post-radiation mucositis which correlates with increases in cytokines (14). However, IL-6 was increased in patients who received surgery alone, suggesting that this increase is related to the response to the treatment, not to the radiation-induced inflammation (8).

The concurrent increase in IL-6 and IL-8 in post-treatment saliva suggests a common regulatory mechanism, such as NF-κB, which plays an important role in the development and progression of HNC; NF-κB-regulated cytokines are upregulated in saliva in HNC (15). The activation of pro-inflammatory cytokines plays a key role in the development of oral mucositis occurring after the first initiation phase, consisting of the generation of reactive oxygen species that promotes upregulation of messenger signals. One of the most important factors is NF-kB, which in turn activates several genes, including those linked to the production of the pro-inflammatory cytokines (12).

Not only radiotherapy treatment but also CTRT seemed to cause a significant increase in IL-1β, IL-6 and TNF-α, all positively associated with the severity of mucosal toxicity. This finding suggests that the elevation of these cytokines three weeks after therapy initiation could be predictive of a more severe oral mucositis. Furthermore, it may represent a potential approach for early identification of risk patients (12).

In several of the cytokines that Citrin *et al.* (5) analyzed, a trend toward increasing levels with increasing the dose delivered was observed for IL-6, IL-8, MCP-1, and TNF-α. Subsequent sampling is likely to provide more striking differences in the levels of cytokines in saliva compared to baseline measurements. To more tissue changes such as inflammation and desquamation due to higher doses of radiation.

The differential changes observed between different cytokines pre- and post-treatment support the idea that the variation is not entirely a function of reduced fluid content of post-treatment saliva, which would increase all cytokines. Moreover, since ionizing radiation increases expression of pro-inflammatory and proangiogenic factors, differences in cytokines between patients treated with radiation or surgery were also investigated by Russo *et al.* (8), suggesting that changes in cytokines did not vary as a function of treatment modalities.

There are limitations to this review that we should highlight. Firstly, not all the studies were comparable as only some of them were case-control studies. Besides, the cytokines were not measured using the same technique. Secondly, the sample size regarding the levels of salivary inflammatory proteins pre- and post-treatment was relatively small. A larger sample cohort would allow for a greater statistical comparison between the two groups.

## Conclusions

Radiation alone or in a combination with chemotherapy is frequently used in the management of malignancies in the head and neck, being the primary treatment option for advanced head and neck cancers. Despite the increasing number of studies and reviews concerning salivary biomarkers for HNC, there is no consensus regarding which biomarker has the best diagnostic value (16). This review demonstrates that screening salivary cytokine levels could provide a useful approach to identifying biomarkers for predicting radiotherapy outcome in HNC. However, more studies are needed to verify the utility of these proteins in prognosis and diagnosis of HNC and as an indicator of the patient’s response to the treatment.
